# Novel combination of feed enzymes to improve the degradation of *Chlorella vulgaris* recalcitrant cell wall

**DOI:** 10.1038/s41598-019-41775-0

**Published:** 2019-03-29

**Authors:** Diogo Coelho, Paula A. Lopes, Vânia Cardoso, Patrícia Ponte, Joana Brás, Marta S. Madeira, Cristina M. Alfaia, Narcisa M. Bandarra, Henri G. Gerken, Carlos M. G. A. Fontes, José A. M. Prates

**Affiliations:** 1 CIISA - Centro de Investigação Interdisciplinar em Sanidade Animal, Faculdade de Medicina Veterinária, Universidade de Lisboa, 1300-477 Lisboa, Portugal; 2 NZYTech - Genes and Enzymes, Estrada do Paço do Lumiar, Campus do Lumiar, Edifício E, 1649-038 Lisboa, Portugal; 3 DivAV, Instituto Português do Mar e da Atmosfera, Rua Alfredo Magalhães Ramalho, 1495-006 Lisboa, Portugal; 4 Arizona Center for Algae Technology and Innovation, Arizona State University, 7418 Innovation Way South, Building ISTB-3, Room 103, Mesa, Arizona United States of America

## Abstract

In this study, a rational combination of 200 pre-selected Carbohydrate-Active enzymes (CAZymes) and sulfatases were tested, individually or combined, according to their ability to degrade *Chlorella vulgaris* cell wall to access its valuable nutritional compounds. The disruption of microalgae cell walls by a four-enzyme mixture (Mix) in comparison with the control, enabled to release up to 1.21 g/L of reducing sugars (p < 0.001), led to an eight-fold increase in oligosaccharides release (p < 0.001), and reduced the fluorescence intensity by 47% after staining with Calcofluor White (p < 0.001). The Mix treatment was successful in releasing proteins (p < 0.001), some MUFA (p < 0.05), and the beneficial 18:3*n*-3 fatty acid (p < 0.05). Even if no variation was detected for chlorophylls (p > 0.05), total carotenoids were increased in the supernatant (p < 0.05) from the Mix treatment, relative to the control. Taken together, these results indicate that this four-enzyme Mix displays an effective capacity to degrade *C. vulgaris* cell wall. Thus, these enzymes may constitute a good approach to improve the bioavailability of *C. vulgaris* nutrients for monogastric diets, in particular, and to facilitate the cost-effective use of microalgae by the feed industry, in general.

## Introduction

Autotrophic microalgae are currently considered an attractive source of high-value chemicals for biofuel, nutraceutical and pharmaceutical industries^1^, as well as sustainable animal production^2^. While the nutritional profile of microalgae varies considerably with the species, a large majority are characterised by having high protein, carbohydrate, lipid, vitamin, mineral and pigment contents^3^, which are comparable, if not superior, to conventional feedstuffs. These alternative feedstuffs are rich in beneficial *n*-3 long-chain polyunsaturated fatty acids (*n*-3 LCPUFA)^4^. The enriched concentration of *n*-3 LCPUFA by microalgae represents a largely untapped natural resource with well-known beneficial health implications for both animals and humans^5^.

*Chlorella vulgaris*, a freshwater unicellular eukaryotic microalga, is one of the most cultivated microalgae worldwide. Although it is known for its relative ease of cultivation and high biomass productivity^6^, *C. vulgaris*, like the majority of microalgae, is endowed with a recalcitrant cell wall that confers resistance against invaders and harsh environmental conditions such as desiccation during growth, and is therefore refractory to breakage and drying, and hence to product extraction^7^. These cell walls have shown to contain an incredibly diverse and complex matrix of cross-linked insoluble carbohydrates, which trap valuable nutrients, thus limiting their direct use. The cell wall structure and composition were recently reviewed by Baudelet *et al*.^1^ for *Chlorellae* genus, and Safi *et al*.^8^ for *Chlorella* species.

Due to their recalcitrant nature, microalgal cell walls are largely indigestible by monogastric animals. For microalgae species, contrary to macroalgae, mechanical methods, as hammer mills, are not usually applied^9^. In turn, bead milling is used to incorporate *Chlorella* cells as food additives and this is a successfully, rising process in the food industry. However, this mechanical process is characterised as being hard working and expensive whereupon cells are massive destroyed. It is therefore imperative to find novel technologies to disrupt *Chlorella vulgaris* cells whereby cell wall disruption would be under a strictly controlled process to improve microalgal nutrient utilization, in particular to gain access to their protein and lipids^2,10^. Another essential prerequisite for the large-scale use of algal biomass as a feed supplement is to achieve a low production cost^6^. In addition, *Chlorella* is also considered as a potential source of microalgae oils for biofuel production and recognised as one of the alternatives to current biofuel crops, such as soybean, corn, rapeseed and lignocellulosic feedstock because it does not compete with food and does not require arable lands to grow^11^. The exploitation of biofuel production by *Chlorella* is thus attracting considerable attention. *Chlorella* has the ability to fix carbon dioxide efficiently and to remove nutrients rich in nitrogen and phosphorous, making it a good candidate for greenhouse gas biomitigation and wastewater bioremediation^3^.

Exogenous Carbohydrate-Active enzymes (CAZymes), mainly xylanases and beta-glucanases, are now widely used to supplement diets of monogastric livestock species to improve feed nutritive value and directly impact on animal performance and health^12^. The use of feed enzymes is currently a cost-effective strategy to improve the nutritional value of cereal-based diets for monogastric animals, although it remains to be established for microalgae biomass. In line with this, we hypothesised that the efficiency of *C. vulgaris* microalgae could be fine-tuned using individually or combined CAZymes and sulfatases, due to the degradation of recalcitrant cell wall and subsequent increase in nutrients bioavailability. Herein, cell disruption induced by enzymatic treatment was assessed by optical and fluorescence microscopies, and by measuring the reducing sugars and the oligosaccharides profile. The release of bioactive compounds with nutritional interest was assessed by quantifying proteins and pigments, as well as the fatty acid content and detailed composition, in both supernatant and residue fractions after incubation with the enzymatic treatment.

## Results

### Individual screening of enzymes in *Chlorella vulgaris* cell wall disruption

In order to evaluate which CAZymes and sulfatases of the library created in this work have the capacity to degrade *C. vulgaris* cell wall, each one of the enzymes was individually incubated with a microalgae suspension. Although a great majority of the enzymes were unable to deconstruct the marine biomass, 29 individual enzymes displayed a measurable capacity to degrade the cell wall of *C. vulgaris*, as described in Table 1. The ability to degrade the microalgae was assessed by the capacity to release reducing sugars as evaluated through the 3,5-dinitrosalicylic acid (DNSA) method. Table 1 data is presented in a qualitative scale of the amount of reducing sugars released (g/L): −, 0.00 < 0.005; +, 0.05 < 0.200; ++, 0.200 < 0.300; +++, >0.300. Although the release of reducing sugars was undetected for four enzymes, with identification numbers (ID) 69, 73, 77 and 82, they were included in this selection because their predicted substrates (1,3-α-glucans; agar and neoagarooligosaccharides; 1,3-β-glucans and insoluble 1,3-β-glucans, respectively) are major constituents of *C. vulgaris* cell walls^8,13^. Within this group of enzymes, CAZymes with ID 36, 47 and 60 exhibited the highest release of reducing sugars from the marine biomass, whereas the remaining enzymes displayed a low to moderate capacity to attack the complex polysaccharides.

**Table 1:** Screening of the selected individual CAZymes-sulfatases and Mix in *Chlorella vulgaris* cell wall disruption.

ID	Name	Category	EC	Main Substrate	Reducing Sugars Released Scale
5	Cellulose 1,4-β-cellobiosidase	Cellobiohydrolases	3.2.1.91	Phosphoric acid-swollen cellulose, Avicel and others forID of insoluble cellulose	++
10	Laccase	Laccases	1.3.3.5	2,20-azinobis(3-ethylbenzthiazoline-6-sulfonic acid) (ABTS)	+
14	Laminarinase	1,3-β-Glucanases	3.2.1.39	1,3-β-glucans such as laminarin	+
16	Chitinase 1	Chitinases & Chitosanases	3.2.1.14	Chitin and chitosan	++
18	Oligoalginate lyase	Alginate lyases	4.2.2.	Low-viscosity alginate	+
25	β-1,3-1,4-glucanase P2	1,3-1,4-β-Glucanases	3.2.1.73	1,3-1,4-β-glucans	+
29	Algal laminarin-specific β-glucanase/laminarinase	1,3-β-Glucanases	3.2.1.39	1,3-β-glucans, such as laminarin, and display low activity on mixed linked glucans	++
32	Endo-β-1,3(4)-glucanase	1,3-1,4-β-Glucanases	3.2.1.6	1,3-1,4-β-glucans, such as lichenan and laminarin.	+
33	β-1,3-glucanase/laminarinase	1,3-β-Glucanases	3.2.1.39	Laminarin	++
36	Chitosanase	Chitinases & Chitosanases	3.2.1.132	Chitosan	+++
37	Endo-β-2,6-fructanase	Fructanases	3.2.1.65	Levans	+
38	Cellobiohydrolase	Cellobiohydrolases	3.2.1.91	Amorphous and crystalline cellulose	+
42	Trans-sialidase B	Sialidases	3.2.1.18	Sialic acids from complex carbohydrates and glycoprotein human alpha-1 (AGP)	++
47	Chitosanase	Chitinases & Chitosanases	3.2.1.132	Soluble and colloidal chitosan	+++
50	α-glucuronidase	Glucuronidases	3.2.1.139	Glucuronic acid from the xylan backbone	+
60	Exo-β-glucosaminidase	Glucosaminidases	3.2.1.165	The 1,4-β-glycosidic bond of cellooligosaccharides, also hydrolysis nonreducing end of chitooligosaccharides (Glc-PNP)	+++
66	Alginate lyase	Alginate lyases	4.2.2.3	Polyguluronate and polymannuronate	+
69	α-1,3-Glucanase	α-Glucosidases	3.2.1.59	1,3-α-glucan	−
73	Exo-β-agarase D	Agarases	3.2.1.81	Agarose and neoagarooligosaccharides	−
77	Endo-β-1,3-glucanase	Laminarinases	3.2.1.39	1,3-β-glucans	−
78	Keratan sulfate hydrolase/keratanase II	Acetylglucosaminidases	3.2.1.103	Cartilage keratan sulfate and cornea keratan sulfate	+
81	Exo-β-glucosaminidase	Glucosaminidases	3.2.1.165	Lactose, GlcNAc2, GlcNAc3, cellobiose, cellotriose, colloidal chitin, cellulose, lichenan, laminarin and xylan	+
82	β-1,3-Glucanase B	Laminarinases	3.2.1.39	Insoluble 1,3-β-glucan	−
85	β-Galactosidase	β-Galactosidases	3.2.1.23	β-galactosides	+
86	Lytic transglycosylase	Peptidoglycan lytic exotransglycosylases	4.2.2.n1	1,4-β-glycosidic bonds between N-acetylmuramic acid and N-acetylglucosamine residues	+
92	Endo-rhamnogalacturonan lyase	Rhamnogalacturonan lyases	4.2.2.23	Rhamnogalacturonan	++
93	Peptidoglycan N-acetylmuramic acid deacetylase	Acetylglucosamine deacetylases	3.5.1.104	Peptidoglycan	+
95	Lysozyme	Lysozymes	3.2.1.17	Peptidoglycans	+
101	Lysozyme (CPE1314)	Lysozymes	3.2.1.17	Peptidoglycan containing muramic acid δ-lactam	+
Mix	**Exo-β-glucosaminidase, Alginate lyase, Peptidoglycan N-acetylmuramic acid deacetylase and Lysozyme (CPE1314) (ID 60, 66, 93 and 101, respectively)**				1.21 g/L

Each enzyme is presented with the identification number (ID), project identification, category, EC number, main substrate and a qualitative scale of reducing sugars released. The enzymatic constitution of the Mix is also presented, as well as the value of the reducing sugars released in g/L. The qualitative scale is based on the amount of reducing sugars released (g/L): −, 0.00 < 0.005;+, 0.05 < 0.200; ++, 0.200 < 0.300; +++, >0.300.

### Composition of a four-enzyme constituted Mix based on reducing sugars released

With the purpose of finding synergistic actions between the individual enzymes identified, the 29 enzymes presented in Table 1 were combined and tested in a mixture for the capacity to release reducing sugars from the microalgae. A mixture (Mix) consisting of four-enzymes was found to be the most restricted combination in terms of enzyme numbers and displaying the highest level of released sugars. This Mix was composed of an exo-β-glucosaminidase, an alginate lyase, a peptidoglycan N-acetylmuramic acid deacetylase and a lysozyme (CPE1314) and is presented in detail in Table 1. When this Mix was incubated with *C. vulgaris* suspension, a value of 1.21 g/L (p < 0.001) of reducing sugars released was observed, which represents an increase of 1.6-fold in relation to the highest value found in the individual enzyme screening. The rates for released sugars were found to be: for Mix *vs* Control = 333.7%; for Mix *vs* exo-β-glucosaminidase = 38.1%; for Mix *vs* alginate lyase = 198.4%; for Mix *vs* peptidoglycan N-acetylmuramic acid deacetylase = 248.1%; for Mix *vs* lysozyme (CPE1314) = 248.1%.

### Thermostability and proteolysis assays

**Figure 1:**
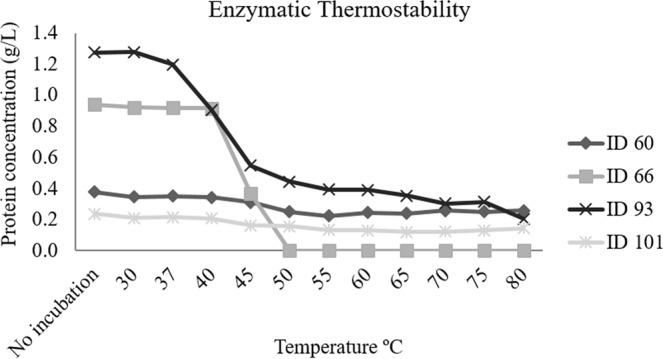
Thermostability analysis for the four enzymes constituting the Mix at different temperatures (30 to 80 °C) and for the control without incubation.

The four enzymes that constitute the Mix treatment were subjected individually to different temperatures to test their thermostability. Figure 1 illustrates the variation of protein concentration across the range of temperatures tested. At 37 and 40 °C, representing the internal temperature of mammals and poultry, respectively, all enzymes maintained their stability. However, the stability of ID 93 and ID 66 decayed abruptly from 37 and 40 °C, respectively. ID 66 even reached complete degradation at 55 °C, while ID 60 and ID 101 remained stable up to 80 °C. To investigate the capacity of the four enzymes to resist to the proteolytic attack, to which feed enzymes are subjected in the animal gastrointestinal tract, the same enzymes were treated with pancreatin at 37 °C. Table 2 shows the proteolytic resistance of these enzymes. ID 60 and ID 101 had partial resistance over the entire assay time; in turn, ID 66 and ID 93 showed complete degradation after 15 minutes.

**Table 2:** Proteolysis resistance for each one of the four enzymes that constitute the Mix. Each enzyme, at a concentration of 1 g/L, was subjected to the proteolytic action of pancreatin, which was incubated at a final concentration of 2.5 g/L.

ID	Time (min)				
	15	30	60	90	120
60	+	+	+	+	+
66	−	−	−	−	−
93	−	−	−	−	−
101	+	+	+	+	+

The reactions were incubated at 37 °C, at regular intervals of 15 min for 120 min. Results are presented at periods of 15, 30, 60, 90 and 120 min of incubation for each enzyme. The qualitative scale on proteolysis resistance is based on SDS-PAGE gels visualisation: −, no resistant (only fragmentation bands); +, partially resistant (protein and fragmentation bands).

### Effect of Mix treatment on *Chlorella vulgaris* cell number and cell wall integrity

**Figure 2:**
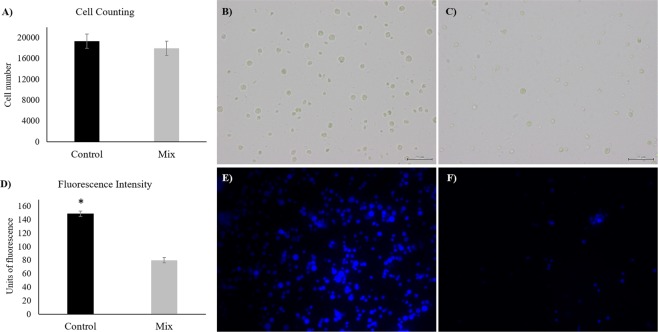
(**A**) Cell counting using a Neubauer chamber for control and Mix treatments. (**B**,**C**) Light microscopy images (×400) of *Chlorella vulgaris* suspension for control and Mix treatments, respectively (scale bar: 20 µm). (**D**) Fluorescence intensity derived from Calcofluor White staining for control and Mix treatments. Asterisk denotes statistical difference at p < 0.001. (**E,F**) Fluorescence images (×400) of *Chlorella vulgaris* suspension stained with Calcofluor White for control and Mix treatments, respectively.

No significant differences were found on the number of cells observed between the control and the Mix (p > 0.05) (Fig. 2A). The number of cells counted was around 20000 cells for both treatments (Fig. 2B,C). The fluorescence intensity was reduced by 47% (Fig. 2D; p < 0.001) when *C. vulgaris* was incubated with the Mix (Fig. 2F), compared to the control (Fig. 2E).

### Effect of Mix treatment on the release of oligosaccharides from *Chlorella vulgaris* cell wall

**Figure 3:**
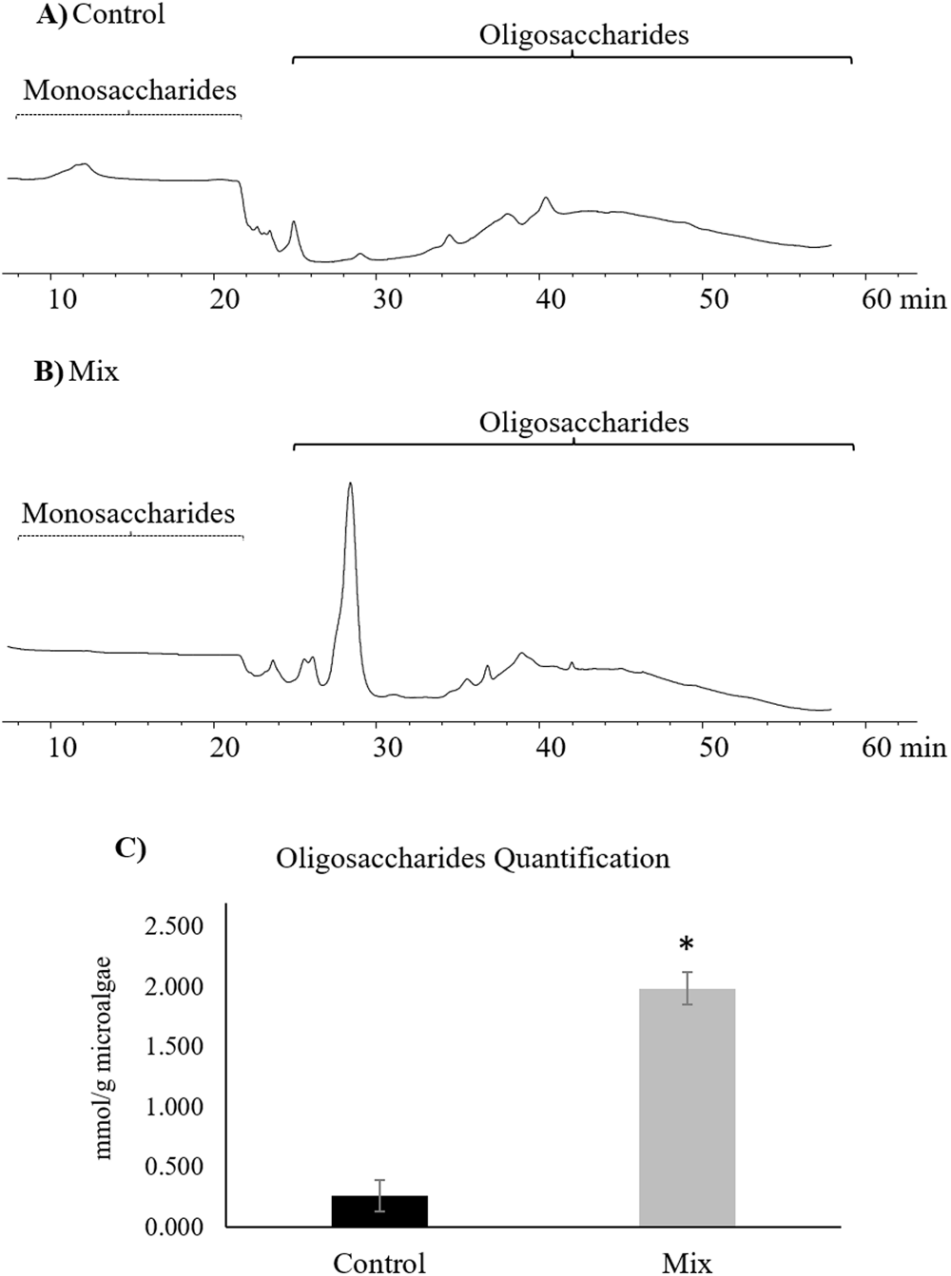
Illustrative chromatogram obtained by the HPLC analysis of supernatants for control (**A**) and Mix (**B**) treatments. Monosaccharides and oligosaccharides regions are shown. The quantification of oligosaccharides is graphically displayed in (**C**). Asterisk denotes statistical difference at p < 0.001.

Figure 3 shows the chromatogram on the release of oligosaccharides from *C. vulgaris* cell wall after treatment with control enzyme mixtures and the Mix enzymes identified in this work. A large peak in the oligosaccharides region was observed in the Mix treatment chromatogram (Fig. 3B) in relation to the control (Fig. 3A), corresponding to an 8-fold increase of oligosaccharides amount (p < 0.001; Fig. 3C).

### Effect of Mix treatment on the release of proteins

In order to understand if the treatment with the Mix triggered the release of proteins from *C. vulgaris* cells to the external environment, the protein content in supernatant and residue fractions was determined (Table 3). In the supernatant, the Mix treatment caused a 23.4-fold increase in protein content relative to the control (p < 0.001), whereas in the residue, the Mix treatment led to a 1.7-fold decrease relative to the control (p < 0.001).

**Table 3:** Content of total proteins, chlorophylls, carotenoids and fatty acids of the supernatant and residue fractions derived from the incubation of *Chlorella vulgaris* with control and Mix treatments.

	Supernatant				Residue			
	Control	Mix	SEM	p-value	Control	Mix	SEM	p-value
Total proteins (mg/g microalgae)	14.6	341.2	17.01	<0.001	776.4	453.8	17.03	<0.001
Chlorophyll a (mg/g microalgae)	0.109*	0.116*	0.006	0.429	2.12**	2.86**	0.301	0.158
Chlorophyll b (mg/g microalgae)	0.154*	0.153*	0.010	0.948	1.27**	2.07**	0.388	0.217
Total chlorophylls (mg/g microalgae)	0.263*	0.269*	0.016	0.799	3.39**	4.93**	0.684	0.187
Total carotenoids (mg/g microalgae)	0.076*	0.083*	0.002	0.032	0.346**	0.268**	0.034	0.185
Total fatty acids (mg/g microalgae)	2.24	2.67	0.417	0.496	23.8	26.4	1.42	0.249
**Fatty acid composition (% total fatty acids)**								
14:0	1.81	1.21	0.216	0.097	1.37	1.30	0.039	0.223
16:0	43.7	44.4	1.59	0.773	23.7	21.8	0.26	0.002
16:1*c*7	0.170	0.512	0.070	0.014	4.17	4.59	0.116	0.043
16:1*c*9	2.17	2.84	0.165	0.028	9.52	11.01	0.226	0.003
17:0	1.72	1.24	0.142	0.053	0.527	0.394	0.044	0.075
17:1*c*9	0.902	3.02	0.307	0.003	6.34	6.53	0.237	0.589
18:0	32.6	27.1	1.04	0.009	7.27	5.17	1.221	0.269
18:1*c*9	6.64	7.56	1.235	0.617	14.3	14.4	0.08	0.291
18:1*c*11	1.79	1.61	0.378	0.741	10.0	9.93	0.277	0.844
18:2*n*-6	3.21	4.96	0.804	0.174	11.3	12.4	0.30	0.041
18:3*n*-6	0.850	0.999	0.220	0.648	0.103	0.127	0.035	0.634
18:3*n*-3	0.719	1.18	0.097	0.015	9.64	11.0	0.385	0.044
20:0	1.59	1.12	0.203	0.155	0.333	0.265	0.036	0.236
22:0	2.19	2.32	0.297	0.769	0.292	0.232	0.024	0.131
22:2*n*-3	nd	nd	—	—	0.430	0.164	0.068	0.033
Others	0.868	0.941	0.199	0.806	0.915	0.778	0.039	0.046
∑ SFA	83.5	77.3	1.94	0.063	33.5	29.2	1.52	0.093
∑ MUFA	11.7	15.6	1.48	0.114	44.3	46.5	0.83	0.116
∑ PUFA	4.78	7.14	0.741	0.065	21.4	23.7	0.67	0.055
∑ *n*-3 PUFA	0.719	1.18	0.097	0.015	10.1	11.2	0.38	0.085
∑ *n*-6 PUFA	4.06	5.96	0.767	0.130	11.4	12.5	0.293	0.033

Two mL of microalgae suspension was incubated with the four enzymes which constitute the Mix at a final concentration of 20 mg/L for each enzyme. The control treatment took the same amount of PBS. Incubations were done overnight at 37 °C and 140 rpm. After incubations, supernatant and residue fractions were separated by centrifugation. Only fatty acids whose percentage was >0.5% are presented; nd, not detected. *Values measured in phosphate buffered saline (PBS); **Values measured after extraction with acetone.

### Effect of Mix treatment on the release of chlorophylls and carotenoids

Following the rationale of the previous point, the release of pigments from *C. vulgaris* cells to the external environment was determined in supernatant and residue fractions (Table 3). No significant variations were observed for chlorophylls (p > 0.05). Total carotenoids displayed significant differences with a 1.1-fold increase in the supernatant fraction of the Mix treatment relative to the control (p = 0.032).

### Effect of Mix treatment on the release of fatty acids

The fatty acid content in residue and supernatant fractions, after incubation with the Mix treatment, was analysed to understand if the activity of Mix in the cell wall favoured the release of fatty acids from *C. vulgaris* cells to the external environment (Table 3). For the supernatant fraction, the predominant fatty acids were saturated fatty acids (SFA) > monounsaturated fatty acids (MUFA) > PUFA > *n*-6 PUFA > *n*-3 PUFA, while for the residue higher MUFA percentages were found, in the following order: MUFA > SFA > PUFA > *n*-6 PUFA > *n*-3 PUFA. In the supernatant, the percentage of 18:0 was increased in the control relative to the Mix treatment (p = 0.009). Conversely, the percentages of 16:1*c*7, 16:1*c*9, 17:1*c*9, 18:3*n*-3 and *n*-3 PUFA were found increased in the Mix treatment in comparison to the control (p = 0.014, p = 0.028; p = 0.003, p = 0.015 and p = 0.015, respectively). For the residue fraction, the Mix treatment presented higher percentages of 16:1*c*7, 16:1*c*9, 18:2*n*-6, 18:3*n*-3 and *n*-6 PUFA (p = 0.043, p = 0.003, p = 0.041, p = 0.044 and p = 0.033, respectively), and lower percentages of 16:0 (p = 0.002) and 22:2*n*-3 (p = 0.033) in relation to the control.

## Discussion

To test the hypothesis that nutrients bioavailability of *C. vulgaris* could be largely improved after disruption of its recalcitrant cell wall, a large library of 178 CAZymes and 22 sulfatases, with well-defined and carefully thought-out enzymatic characteristics, was established by recombinant expression in *E. coli* cells. These 200 enzymes were selected taking into account the composition of the known matrix polysaccharides of microalgae cell walls, which comprises pectin, chitin agar, alginates or the aliphatic polymer algenan^14^. The selected enzymes were produced in a high-throughput (HTP) platform that involves gene synthesis, gene cloning, protein expression and protein purification. These enzymes were screened individually to degrade *C. vulgaris* cell wall, which was firstly assessed by measuring the release of reducing sugars. In the next stage, the 29 recombinant enzymes able to degrade *C. vulgaris* cell wall (see Table 1) were tested in combination to obtain the maximum disruption of *C. vulgaris* cell wall. As a result of these combinations, a four-enzyme mixture (Mix) was identified as the most active in the degradation of *C. vulgaris* cell wall and applied throughout.

The selected Mix was composed of four recombinant enzymes, an exo-β-glucosaminidase, an alginate lyase, a peptidoglycan N-acetylmuramic acid deacetylase and a lysozyme. The exo-β-glucosaminidase, included in the category of glucosaminidases^15^, has cellooligosaccharides and chitooligosaccharides as main substrates. The alginate lyase belongs to the family 5 of PL and has polyguluronate and polymannuronate as main substrates^16–18^. The peptidoglycan N-acetylmuramic acid deacetylase, which is included in the category of acetylglucosamine, has peptidoglycan as main substrate^19^. Finally, lysozyme, also known as muramidase belongs to GH family 25^16^ and peptidoglycan containing muramic acid δ-lactam is the main substrate of this enzyme^20^. The four enzymes constituting the Mix were biochemically characterised in terms of their thermostability and resistance to proteolysis. Both ID 60 and ID 101 were stable throughout the range of temperatures tested and resistant to the proteolytic action of pancreatin. The tertiary structure of protein, which confers thermotolerance to enzymes, could also confer inherent proteinase resistance, as demonstrated by Fontes *et al*.^21^. In contrast, enzymes ID 66 and ID 93 were shown to be sensitive to temperature rise and to proteolysis.

With the aim of evaluating the capacity of enzymes to digest the *C. vulgaris* cell wall for lipid extraction, Gerken *et al*.^22^ focused on the inhibition of *C. vulgaris* growth by a variety of enzymes. *C. vulgaris* is typically sensitive to chitinases and lysozymes, both enzymes degrading polymers containing N-acetylglucosamine. This observation corroborates our results with the introduction of a lysozyme, a glucosaminidase and an acetylglucosamine deacetylases in the Mix. Even if the composition of *C. vulgaris* cell wall is not entirely known, it is formed by a complex matrix constituted by glucosamine or galactose and mannose, and a broad range of pentose and hexose sugars^23^. As discussed by Baudelet *et al*.^1^, certain viruses can infect *C. vulgaris*, digest the host cell wall, penetrate and let the newly synthesised virus to be released^24,25^. Baudelet *et al*.^1^ referenced the identification of cell wall degrading alginate lyase coding genes in the genome of *C. vulgaris* infecting virus. These insights remit to the importance of alginate lyase for *C. vulgaris* cell wall digestion.

The Mix was proven effective by the increase in reducing sugars released, indicating a likely synergistic effect of these enzymes, as described by Phong *et al*.^26^, and known when enzyme mixes degrade carbohydrate mixtures. Fu *et al*.^27^ with the aim to evaluate the capacity of an immobilised cellulase to hydrolyse the cell wall of *Chlorella* sp. under different conditions also used the measurement of reducing sugars. As *Chlorella vulgaris* has been described as having a residual content of polysaccharides inside the cell^27^, the oligosaccharides found came from the disruption of cell wall instead of cell interior. Moreover, when applying the enzymatic mixture treatment to *Chlorella vulgaris* cells, it is expected that the first structure to be affected and partially or entirely disrupted would be the cell wall with the concomitant release of reducing sugars, easily measurable by the 3,5-dinitrosalicylic acid method.

No significant differences were observed in *C. vulgaris* cell number after treatment with the four-enzyme Mix. However, the fluorescence intensity was reduced by 47% with the Mix, indicating that these exogenous enzymes do not lead to the complete degradation of the cell wall, although cell wall integrity was affected to a major degree. Safi *et al*.^28,29^ used the same fluorochrome on different species of microalgae, including *C. vulgaris*, before and after different cell wall disintegration methods (*e.g*. high-pressure and bead milling) were applied. This clear change in cell structure was reinforced in our study by an increase of oligosaccharides amount after the treatment with the Mix, as reported by Heo *et al*.^30^. Those authors observed a dramatic increase of glucose content in *C. vulgaris* after an osmotic shock treatment, which was related to an efficient cell wall disruption. Conversely, in our study, there was no complete degradation of carbohydrates from the cell wall, since a complex mixture of oligosaccharides rather than single sugars was obtained.

The Mix treatment effect on *C. vulgaris* cell wall was a massive release of (hydro-) soluble proteins found in the supernatant, which was counterbalanced by a considerable decrease of proteins in the residue. These results agree with Safi *et al*.^28,29^, who observed an increase in soluble protein concentration after the application of different mechanical and chemical cell wall disruption methods in *C. vulgaris*. *C. vulgaris* has a high protein content, up to 68%^3^, with great nutritional quality since its amino acid composition meets the human dietary requirements proposed by World Health Organization (WHO) and Food and Agricultural Organization (FAO)^8^.

The Mix treatment also promoted the beneficial release of total carotenoids to the supernatant. Carotenoids, in particular β-carotene, astaxanthin, cantaxanthin and lutein, have various therapeutic properties, such as prevention of retina degeneration^31^ and regulation of blood cholesterol^32^, which are associated with their antioxidant activity^33^ and account for 1% in *C. vulgaris*^29,34^. Chlorophyll is the most abundant pigment in *C. vulgaris*, reaching 1–2% of the microalga dry weight. Even though the Mix treatment promoted the release of total carotenoids to the supernatant, no variation was observed for chlorophylls a and b. Safi *et al*.^29^ showed that the disruption of cell wall through the application of several mechanical and chemical methods allowed to release chlorophylls and carotenoids to the aqueous phase^29^, and at least for carotenoids, their results concur with ours. We speculate that the Mix treatment was unable of penetrating the phospholipid bilayer of the chloroplast in which pigments, such as chlorophylls and primary carotenoids, are embedded inside the thylakoids, therefore justifying the absence of differences for chlorophylls^29^. Alternatively, the presence of chlorophylls and carotenoids in the supernatant indicates the formation of micellar structures^29^, which are in line with their amphiphilic characteristics sharing different degrees of polarity^35^.

The fatty acid content and composition described herein for *C. vulgaris* cells agree with previous reports^36,37^, regardless the enzymatic treatment. Several studies, including those of Heo *et al*.^30^, Zheng *et al*.^36^, Cho *et al*.^38^ and Liang *et al*.^39^, were performed to improve the yield of lipid extraction from microalgae. A substantial cell wall disruption was observed by Cho *et al*.^38^ using a mixture of cellulases and β-glucosidases, and by Zheng *et al*.^36^ using a mixture of snailase, lysozyme and cellulase. In both cases, the enzymatic treatment led to an increase in lipid extraction efficiency, highlighting in the case of Zheng *et al*.^36^, the good performance exhibited by lysozyme. In our study, the focus was not on whether the Mix led to an increase in lipid extraction yield but, instead, on the release of fatty acids from *C. vulgaris*, through the disruption of microalgae cell wall. The major differences were found at the level of some MUFA, with a higher release of 16:1*c*7, 16:1*c*9 and 17:1*c*9, when *C. vulgaris* was submitted to the Mix treatment, justifying a higher percentage in the corresponding supernatant. The same applies to α-linolenic acid (18:3*n*-3), an essential *n*-3 LCPUFA, with important health properties, in particular for the prevention of cardiovascular diseases, cancer, autoimmune diseases and type 2 diabetes^5,40^. Due to its benefits, the increase release of α-linolenic acid when using this Mix deserves to be further exploited.

## Conclusion

The results reported in this work indicate that this four-enzyme Mix has capacity to partially degrade *C. vulgaris* cell wall. These findings open new opportunities to develop a novel generation of biocatalysts to supplement diets for monogastric animals, in particular those incorporating *C. vulgaris* microalga. Data indicate that exogenous enzymes may disrupt microalgae cell walls to a significant extent, allowing the release of trapped nutrients with important nutritional value. Consequently, exogenous enzymes may promote the use of microalgae in animal diets at higher incorporation levels (>1%), leading to the release of highly beneficial bioactive compounds in an economically viable way. Further work is ongoing at our research laboratories to assess how effective these combined enzyme activities are for the supplementation of monogastric diets with *C. vulgaris* microalga as a feed ingredient. In addition to the animal feed industry, these results may increase the yield in obtaining valuable constituents of *C. vulgaris* for other biotechnological industries, in particular those related with biofuel, food and nutraceutical applications.

## Methods

### Microalgae production

*Chlorella vulgaris* is an unicellular freshwater microalgae of the genus *Chlorella* characterised by a relative ease of cultivation, high productivity and high content of proteins, lipids and other valuable components^6^. It has emerged as a promising alternative feedstock that represents an enormous biodiversity with multiple benefits exceeding the potential of conventional agricultural feedstock^8^.

*C. vulgaris* was cultivated through inoculation of axenic microalgal cultures (from the Institutes algal banks) in a medium that stimulates the growth of *C. vulgaris:* NaNO_3_ (250 mg/L), KH_2_PO_4_ (105 mg/L), MgSO_4_ (75 mg/L), CaCl_2_ (25 mg/L), NaCl (25 mg/L), K_2_HPO_4_ (75 mg/L), and 3 mL of trace metal solution: FeCl_3_ (0.194 g/L), CoCl_2_ (0.16 g/L), MnCl_2_ (0.082 g/L), Na_2_MoO_4_·2H_2_O (0.008 g/L) and ZnCl_2_ (0.005 g/L), using the adapted Krauss medium^41^.

*C. vulgaris* was first grown in 1 L capacity airlift bioreactors and then scaled-up until 25 L capacity polyethylene bag bioreactors (40 cm diameter) with bubbling filtered air (without CO_2_ addition), at low incident light conditions (150 µE.m^−2^.s^−1^), and at the optimal temperature of 25 °C for *C. vulgaris*. The harvesting step was done after reaching the stationary growth phase. Microalgal biomass was harvested without flocculation by simply removing agitation, followed by centrifugation in a continuous centrifuge LPX 40 (Alfa Laval, Sweden) (25 L). The concentrated biomass slurry was then frozen at −20 °C and freeze dried (Powerdry LL 3000, Thermo, Denmark) for further analysis.

### High-throughput gene synthesis, cloning and protein expression/purification of recombinant enzymes

One-hundred and seventy-eight CAZymes with high potential for degradation of microalgae cell wall were selected from a diverse repertoire, including glycoside hydrolases (GH), pectate lyases (PL) and carbohydrate esterases (CE). In addition, twenty-two sulfatases were also selected for screening, as they are also likely involved in microalgae cell wall degradation^22^. The coding genes for the selected enzymes were synthesised *in vitro* using NZYGene Synthesis kit (Nzytech, Portugal). The protein sequence of each enzyme is presented as Supplementary Material (Table S1). Synthetic genes were codon optimised for expression in *Escherichia coli*, using NZYTech′s codon optimization software ATGenium^42^. All genes included the required 16 bp overhangs on both 5′ and 3′-ends for direct cloning into the bacterial expression vector pHTP1 (Nzytech, Portugal), following the procedure described in the NZYEasy Cloning & Expression kit I (Nzytech, Portugal). The generated recombinant plasmids were subjected to inducible T7 promoter control, while encoding the 200 enzymes fused to an N-terminal His6-tag that facilitates purification through Immobilised Affinity Chromatography (IMAC). The two-hundred plasmids were sequenced to ensure that no mutations accumulated during gene synthesis and were used to transform *E. coli* BL21 (DE3) cells. Transformed cells were grown on solid media and resulting colonies were used to inoculate 5 mL of NZY Auto-Induction LB medium (Nzytech, Portugal) supplemented with kanamycin (50 mg/L) at 37 °C to early-exponential phase (A600 nm = 1.5–2.0). Recombinant protein production occurred following a further incubation at 25 °C for 16 hours. All steps were carried out in 24 deep-well plates^42^. Cells were harvested by centrifugation at 75,000 *g* at 4 °C for 15 min and lysed in NZY Bacterial Cell Lysis Buffer (NZYTech, Portugal). The His6-tagged recombinant enzymes were purified from cell-free extracts by IMAC, based on an automated protocol that allows the purification of 96 proteins by day, as described previously^43^. Briefly, the crude cell lysates were incubated with Sepharose chelating beads (200 μL with bound Ni^2+^) and then transferred into 96-well filter plates (Macherey-Nagel). The wells were washed twice with buffer A (50 mM Na-HEPES, pH 7.5, 500 mM NaCl, 10 mM imidazole). The recombinant fusion proteins were eluted from the resin beads with 200 μL of elution buffer (50 mM Na-HEPES, pH 7.5, 500 mM NaCl, 300 mM imidazole) into 96-deep-well plates. All protein purification steps were automated on a Tecan robot (Tecan, Switzerland) containing a vacuum manifold. Homogeneity of purified proteins and molecular mass of recombinant enzymes were determined by SDS-PAGE in 14% (w/v) acrylamide gels. Protein concentration of enzymes stock solutions varied between 0.5–20 g/L, as determined spectrophotometrically by the Bradford method^44^.

### Preparation of microalga cells suspension

*Chlorella vulgaris* suspension was prepared at 20 g/L, as follows: dry microalgae were weighed, re-suspended in phosphate buffered saline (PBS) and incubated for 30 min at 37 °C in an orbital incubator shaker at 200 rpm. After incubation, the suspension was centrifuged at 2500 *g* for 30 min, the supernatant was discarded and the pellet re-suspended in the same amount of PBS.

### Enzymatic cell wall disruption

The cell wall disruption assay was performed in a 24 well microplate (VWR Chemicals, West Chester, PA, USA). Two mL of microalgae suspension was added to each well along with the respective enzyme added at a final concentration of 20 mg/L. Control wells took the same amount of PBS. The microplate was then sealed and incubated overnight in an orbital incubator shaker at 37 °C and 140 rpm. Microplate was centrifuged for 15 min at 3210 *g* and the supernatants and pellets were recovered. To precipitate and remove the enzymes, the supernatant for DNSA and HPLC analyses was boiled for 5 min and centrifuged for 5 min at 10,000 *g* and the supernatant recovered.

### Reducing sugars measurement

3,5-Dinitrosalicylic acid (DNSA) method^45^, was employed as a standard protocol to quantify the released amount of reducing sugars. In this method, the aldehyde and ketone groups of the reducing sugars reduce the yellow 3,5-dinitrosalicylic acid to orange 3-amino-5-nitrosalicylic acid. Glucose was used as standard. After mixing 0.6 mL of glucose solutions or supernatants with 0.6 mL of DNSA reagent, samples were heated at 100 °C for 15 min. Then, samples were cooled on ice for 5 min and detected by ultraviolet-visible spectrophotometry at 570 nm.

### Thermostability and proteolysis experiments

Each enzyme from the four-enzyme constituted mixture (Mix; Provisional Patent number 20181000067928, INPI, Portugal) was subjected individually to 12 different temperature conditions (without incubation and with incubation at 30 °C, 37 °C and 40 °C to 80 °C at 5 °C intervals) for 30 min. Then, the incubation was cooled on ice for 10 min and centrifuged at 16,100 *g* for 8 min at 4 °C. The supernatant was recovered and the protein amount was quantified in triplicate using a NanoDrop 2000/2000c (NanoDrop Technologies; Thermo Fisher Scientific, Inc., Pittsburgh, PA, USA). To validate results, the supernatants were also analysed by 14% SDS-PAGE gels and the images were acquired with BioRad ChemiDoc XRS imaging system (Bio-Rad, Hercules, CA, USA).

Two-hundred microliters of each enzyme that compose the Mix, at a concentration of 1 g/L, was placed in individual tubes. For each enzyme, there was a control and a treatment: 200 µL of PBS was added for the control; 200 µL of porcine pancreatin (VWR Chemicals, West Chester, PA, USA) at 5 g/L was added for the treated sample. The reactions were incubated at 37 °C, at regular intervals of 15 min until 2 h. The samples were then removed and analysed by 14% SDS-PAGE gels to validate results. The images from gels were acquired with BioRad ChemiDoc XRS imaging system (Bio-Rad). A qualitative scale was created to assess the proteolytic resistance based on the visualisation of protein fragments in SDS-PAGE gels. The qualitative scale was defined as follows: −, no resistant (only fragmentation bands) which means the complete disappearance of the protein band along with visualisation of protein fragments from enzymatic digestion; + , partially resistant (protein and fragmentation bands) meaning that the protein band is visualised associated with protein fragments bands from enzymatic digestion.

### Determination of total oligosaccharides

The mono and oligosaccharide profiles from the supernatants of *C. vulgaris* after control and Mix treatments were analysed by High Performance Liquid Chromatography (HPLC) on an Agilent system (Agilent 1200 Series, Agilent Technologies Inc., Palo Alto, CA), equipped with an electrochemical detector (Coulochem III, ESA Dionex Thermo Fisher Scientific Inc, USA). The HPLC analysis was performed using a Dionex CarboPac PA10 column (4 × 250 mm, Thermo Fisher Scientific Inc, USA) fitted to a CarboPac PA10 guard column (4 × 50 mm), following the procedure described by Thermo Fisher Scientific^46^ with slight modifications. The separation of mono and oligosaccharides was achieved using a mobile phase with a flow rate of 1 mL/min for 60 min at 25 °C, as follows: isocratic elution with 18 mM NaOH (eluent A) during 18 min, gradient with 100–0 mM NaOH (eluent B) and 0–75 mM sodium acetate in 100 mM NaOH (eluent C) from 18–40 min, and re-equilibration to 18 mM NaOH during 20 min. The quantification of total oligosaccharides was based on a standard curve, using a range of concentrations from 0.025 mM to 0.2 mM of glucose. The results were expressed as equivalent moles of glucose released *per* gram of microalgae.

### Optical and fluorescence microscopic observations

The pellets from the enzymatic cell wall disruption assay were re-suspended in 2 mL of PBS. The number of cells in the microalgae suspension was determined using a Neubauer counting chamber by direct observation on a bright-field Olympus CH30 microscope (Olympus, Center Valley, PA, USA) and images were acquired with an Olympus DP21 (Olympus) digital camera. The fluorochrome Calcofluor White (Sigma-Aldrich, St. Louis, Mo, USA) that binds to the cell wall^28^ was added to the same suspensions used for optical microscopy. Cells were observed with an epifluorescence microscope and images were captured with a Leica DFC-340FX (Leica, Wetzlar, Germany) camera system. When excited at λ = 488 nm, cells were identified as blue coloured. The fluorescence intensity, expressed as arbitrary units, was measured using the Image J software^47^.

### Determination of protein content

The N content in lyophilised supernatants and residues from *C. vulgaris* suspension after control and Mix treatments was determined by the Kjeldahl method (984.13)^48^, assuming that no nitrogen from the media interfere with the assay. The crude protein was calculated as N × 6.25.

### Pigment analysis

The content of chlorophyll a, chlorophyll b and total carotenoids in supernatants and residues from *C. vulgaris* suspension after control and Mix treatments was measured according to Hynstova *et al*.^49^, with slight modifications. For the pigment determination in the residue fraction, 4 mL of acetone was added to 40 mg of residue and incubated in the dark during 1 h at 45 °C and 200 rpm. After incubation, the samples were analysed using UV–Vis spectrophotometer (Ultrospec 3100 *pro*, Amersham Biosciences, Little Chalfont, UK) and the pigment content was calculated according to equations described by Hynstova *et al*.^49^. The supernatant fraction was red directly after treatment using UV–Vis spectrophotometer and the pigment content was calculated as defined above.

### Determination of fatty acid composition

Fatty acids from the lyophilised supernatants and pellets of *C. vulgaris* after control and Mix treatments were extracted based on the method of Folch *et al*.^50^, replacing chloroform:methanol (2:1, v/v) by dichloromethane:methanol (2:1, v/v), according to Carlson^51^. Fatty acids were esterified to methyl esters (FAME) by acid catalysis with acetylchloride-methanol solution (1.25 M Sigma-Aldrich, St. Louis, Mo, USA) at 80 °C for 60 min as described by Batista *et al*.^52^. The analysis of FAME was done using a gas chromatograph HP7890A (Hewlett-Packard) coupled with a flame ionization detector (GC-FID). The separation was performed in a SupelcowaxTM^10^ capillary column (30 m × 0.20 mm i.d., 0.20 μm film thickness; Supelco Inc., Bellefonte, PA) with helium as a carrier gas at a flow rate of 1.3 mL/min. The injector and detector temperatures were 250 and 280 °C, respectively. The oven temperature program was started at 150 °C and held for 11 min, then increased to 210 °C at a rate of 3 °C/min and maintained for 30 min. The identification of FAME was achieved by comparison with retention times of fatty acids standards (37 Component FAME mixture from Supelco Inc.). The quantification of total FAME was carried out using heneicosanoic acid (21:0) as internal standard. Each fatty acid was expressed as a percentage of the sum of identified fatty acids (% total fatty acids).

### Statistical analysis

All experiments were conducted in triplicate, and the mean values are presented. The error bars on figures indicate the standard error. Data were checked for normality and analysed using the Generalised Linear Mixed (GLM) model test. p value lower than 0.05 was considered to be statistically significant. All statistical analyses were performed with SAS version 9.4 (SAS Institute Inc., Cary, NC, USA).

## Supplementary information

Supplementary Material S1

## Data Availability

All data generated during this study are included in this published article. The datasets generated during the current study are available from the corresponding author on demand.
